# Exploring the binding sites and proton diffusion on insulin amyloid fibril surfaces by naphthol-based photoacid fluorescence and molecular simulations

**DOI:** 10.1038/s41598-017-06030-4

**Published:** 2017-07-24

**Authors:** Nadav Amdursky, M. Harunur Rashid, Molly M. Stevens, Irene Yarovsky

**Affiliations:** 1 Department of Materials, Department of Bioengineering and Institute of Biomedical Engineering, Imperial College London, London, SW7 2AZ United Kingdom; 2 School of Engineering, RMIT University, Melbourne, Victoria 3001 Australia; 3 Schulich Faculty of Chemistry, Technion - Israel Institute of Technology, Haifa, 3200003 Israel

## Abstract

The diffusion of protons along biological surfaces and the interaction of biological structures with water are fundamental areas of interest in biology and chemistry. Here, we examine the surface of insulin amyloid fibrils and follow the binding of small molecules (photoacids) that differ according to the number and location of their sulfonic groups. We use transient fluorescence combined with a spherically-symmetric diffusion theory to show that the binding mode of different photoacids determines the efficiency of proton dissociation from the photoacid and the dimensionality of the proton’s diffusion. We use molecular dynamics simulations to examine the binding mode and mechanism of the photoacids and its influence on the unique kinetic rates and diffusion properties of the photoacid’s dissociated proton, where we also suggest a proton transfer process between one of the photoacids to proximal histidine residues. We show that the photoacids can be used as fluorescent markers for following the progression of amyloidogenic processes. The detailed characterisation of different binding modes to the surface of amyloid fibrils paves the way for better understanding of the binding mechanism of small molecules to amyloid fibrils.

## Introduction

The etiology of numerous neurodegenerative and non-neuropathic diseases including Alzheimer’s disease, Parkinson’s disease, Huntington’s disease, type II diabetes, and cataracts are related to the formation of amyloid fibrils^1, 2^. During the fibrillogenesis process, the soluble amyloid proteins aggregate into an insoluble structure, which typically consists of a cross-β structure^3^. Significant scientific effort has focused on elucidating the structure of amyloid fibrils, mainly to design molecular inhibitors to fibrillogenesis^4–7^. In order to better explore the fibrillogenesis process, it is of prime importance to understand the surface structure of amyloid fibrils and their interaction with the surrounding aqueous environment. Here, we explore the surface and binding sites of amyloid fibrils formed by the aggregation of amyloidogenic insulin hormone^8–10^ by utilizing the fluorescence of 2-naphthol-based photoacids^11–13^ and all-atom molecular dynamics (MD) simulations^14, 15^.

Photoacids are molecules that have different p*K*_*a*_ values between their electronic ground and excited states – the p*K*_*a*_ value in the excited state (p*K*_a_^*^) is significantly lower than the value in the ground state. Stronger photoacids have lower p*K*_a_^*^ values, and a larger p*K*_*a*_ − p*K*_a_^*^ (Δp*K*_*a*_) difference. Following photoexcitation, the photoacid undergoes the following photoprotolytic cycle in its excited state:

1 $${{\rm{ROH}}}^{\ast }\underset{\mathop{\to }\limits_{{k}_{a}}}{\overset{{k}_{PT}}{\to }}[{{\rm{RO}}}^{\ast }\,\ldots {{\rm{H}}}^{+}]\underrightarrow{\mathop{\to }\limits^{\mathop{\to }\limits_{}}}{{\rm{RO}}}^{\ast }+{{\rm{H}}}^{+}$$

The excited protonated photoacid (ROH^*^) dissociates with a proton transfer rate constant of *k*_PT_ to form an ion pair with the proton. The proton can then either geminate recombine with the deprotonated excited photoacid (RO^−*^) with a rate constant of *k*_a_ or diffuse from the photoacid according to the Debye-Smoluchowski diffusion equation^16^. Usually in aqueous solutions, the water molecule serves as the proton acceptor. This excited-state proton transfer of photoacids has been extensively studied both experimentally and theoretically^11, 17–20^. Since the ROH^*^ and the RO^−*^ forms of photoacids have different emission wavelengths, their steady-state and time-resolved populations can be followed in a facile manner. Using these techniques, the interaction of water with several biological surfaces/binding sites has been studied, such as the interactions with amyloid fibrils^21–26^. In this context it was also shown that the hydrogen-bonds network of amyloid fibrils can behave as a fluorophore by itself having a photoacidity behaviour^27^. In this study, we measure the steady-state and time-resolved fluorescence of various 2-naphthol-based photoacids^11–13^ with different strengths of photoacidity to explore: (i) various binding sites along the insulin amyloid fibril; (ii) the propagation of protons along the fibril surface; and (iii) the interaction of the binding site and its surrounding fibril surface with water.

## Results and Discussion

**Figure 1:**
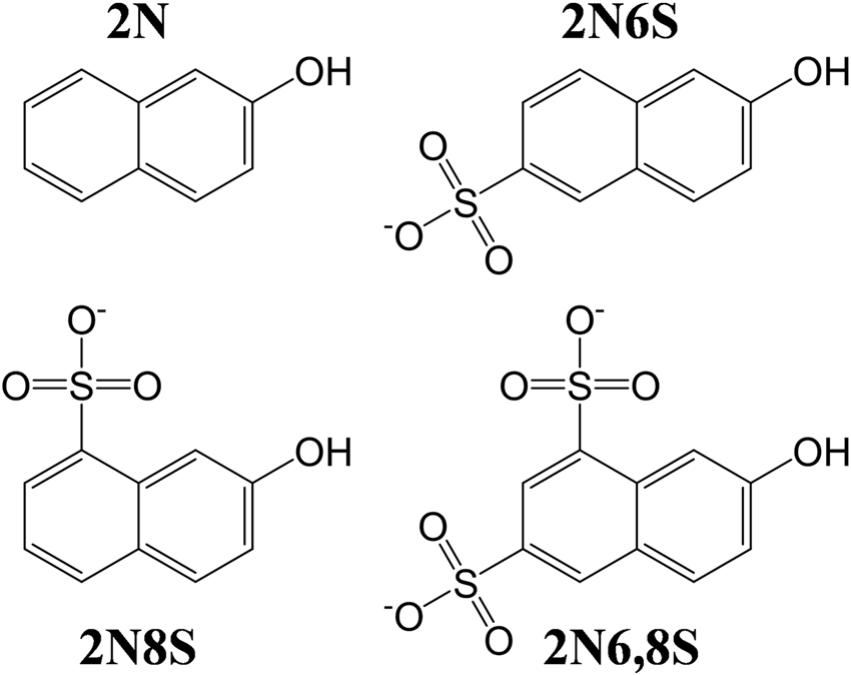
Molecular structure of 2N, 2N6S, 2N8S and 2N6,8S.

The 2-naphthol-based photoacids that we used in this study are (Fig. 1): 2-naphthol (2N), 2-naphthol-6-sulfonate (2N6S), 2-naphthol-8-sulfonate (2N8S), 2-naphthol-6,8-disulfonate (2N6,8S). These photoacids contain a hydrophobic core (the naphthalene ring), which we predict can interact with the fibril hydrophobic amyloidogenic region, and a hydroxyl and sulfonic groups (2N does not have a sulfonic group), which permits the solubility of the photoacid in aqueous solution at the concentration used (45 μM). While all of these photoacids have similar ground state p*K*_*a*_ values in the range of 9–9.5, they differ greatly in their excited state p*K*_a_^*^ values with values of 2.8, 1.7, 1.0 and 0.4 for 2N, 2N6S, 2N8S and 2N6,8 S, respectively. Hence, while 2N is considered a weak photoacid, 2N6,8S is a strong one, having nearly 9 orders of magnitude difference in its *K*_*a*_ values (Δp*K*_*a*_ ≈ 9). In general, stronger photoacids will dissociate upon light excitation (during their excited-state) more efficiently (high *k*_PT_ values) than weak photoacids.

### Steady-state emission

**Figure 2:**
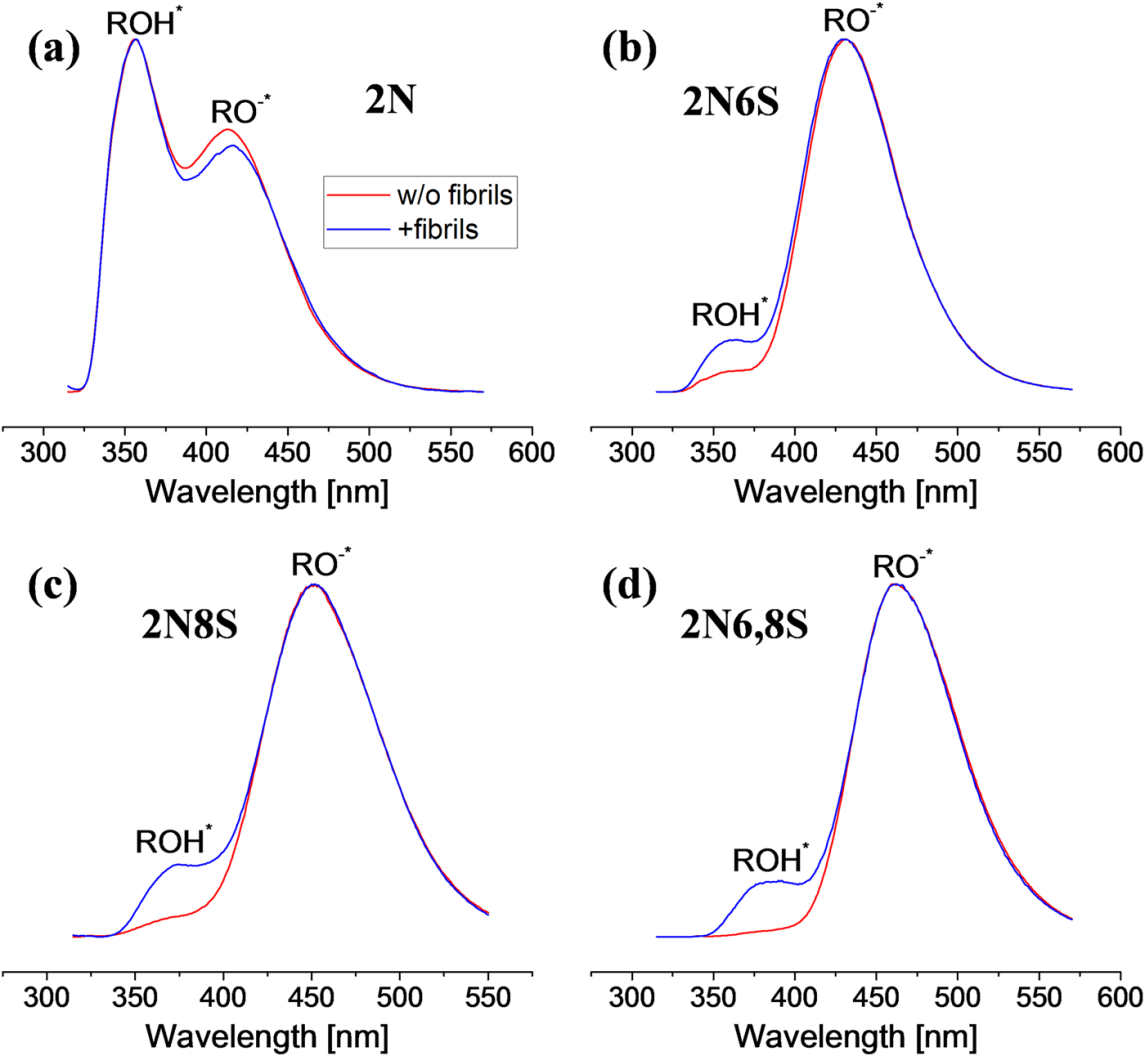
Normalized steady-state fluorescence of the different photoacids: (**a**) 2N, (**b**) 2N6S, (**c**) 2N8S and (**d**) 2N6,8S, in pH7 buffer (red curves), and in the same buffer with insulin fibrils (blue curves).

The proton dissociation rates of the different photoacids as a function of their strengths can be easily observed by their steady-state fluorescence (red curves in Fig. 2). As stated above, stronger photoacids will dissociate more efficiently in aqueous solution, making RO^−*^ the predominant species in the excited-state (Eq. 1), while ROH^*^ is the predominant species in the excited-state of weak photoacids. Accordingly, the RO^−*^/ROH^*^ ratio will increase as a function of the strength of the photoacids. While dissolving the photoacids in pH7, we observed the following ratios from the steady-state spectra: 0.74, 16.6, 18.2 and 48.5 for 2N, 2N6S, 2N8S and 2N6,8S, respectively, a sequence which follows the strength of the photoacid. The steady-state spectrum of 2N (Fig. 2a) also shows that this photoacid is considerably weaker compared to other photoacids studied here since even in the excited-state the predominant species is ROH^*^.

The addition of insulin fibrils to the solution induces the binding of the photoacid to the fibrillar structure, which subsequently induces a change in its excited-state proton dissociation process. As shown previously for the binding of pyranine to insulin fibrils^21^, this binding lowers the steady-state RO^−*^/ROH^*^ ratio of the photoacid. Similarly, the binding of 2N6S, 2N8S and 2N6,8S to the fibrillar structure lowered their ratios to 6.5, 4.9 and 6.4, respectively. Unlike the relatively large change in the RO^−*^/ROH^*^ ratio for the latter photoacids, this ratio for the 2N photoacid hardly changed following the addition of insulin fibrils to the solution (ratio changed from 0.74 to 0.70). Nevertheless, and even though we did not observe a significant change in the RO^−*^/ROH^*^ ratio, we observed a small bathochromic shift in the RO^−*^ band for 2N, which indicated the change in the photoacid polarity following binding to the fibrillar structure^28^.

Although it is clear from the steady-state emission of 2N6S, 2N8S and 2N6,8S that the photoacid can bind to the insulin fibril, we cannot distinguish from the steady-state results whether the change in the RO^−*^/ROH^*^ ratio is due to a slower proton transfer rate (*k*_PT_) of the photoacid when bound to the fibril structure or due to an efficient geminate recombination rate (*k*_a_). The steady-state results of 2N do not strongly indicate the binding of this photoacid to the fibril structure, even though the bathochromic effect suggests it. However, the small change in the RO^−*^/ROH^*^ ratio of 2N is not necessarily an indication of a weak binding between the photoacid and fibril, and it may also be the result of an increase in both the proton transfer rate and geminate recombination rate (or other parameters, see below).

### Time-resolved emission

**Figure 3:**
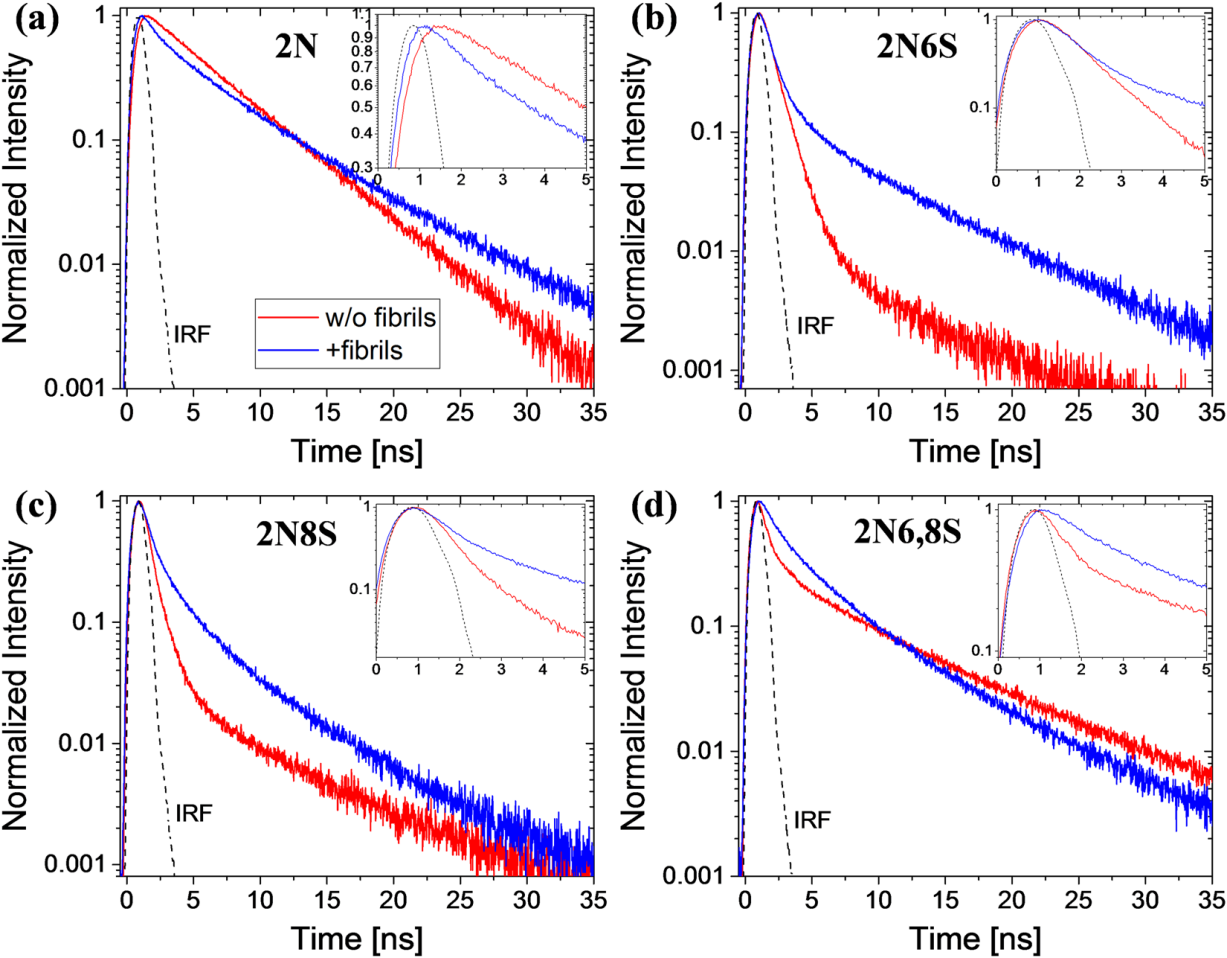
Time-resolved fluorescence of the ROH^*^ band of the different photoacids: (**a**) 2N, (**b**) 2N6S, (**c**) 2N8S and (**d**) 2N6,8S, in pH7 buffer (red curves), and in the same buffer with insulin fibrils (blue curves), together with the instrument response function (IRF, dashed black curves). The graphs are on a semi-logarithmic scale and the insets show a magnification of the first 5 ns. The linear scale representation is in Figure S1.

In order to better explain the observed changes in the steady-state emission of the photoacids, we measured the time-resolved emission of the ROH^*^ band before and after binding of the photoacid to the fibrils (red and blue curves, respectively, in Fig. 3 on a semi-logarithmic scale and in Figure S1 on a linear scale). In general, the decay of the ROH^*^ band of photoacids in aqueous solution is composed of a fast proton transfer decay (*τ*_PT_ = 1/*k*_PT_), which can be observed in the first nanoseconds, followed by a slow geminate recombination decay (*τ*_a_ = 1/*k*_a_), which manifests as a slow tail in the longer timescales. From the decay of the ROH^*^ band, we could qualitatively conclude that the binding of 2N6S, 2N8S and 2N6,8S to insulin fibrils lowered the proton transfer rate of the photoacids. The results of 2N (Fig. 3a) were notable: by contrast to the steady-state measurements, the time-resolved measurements clearly showed that the photoacid was bound to the fibrillar structure due to a fundamentally different decay profile before and after binding to the fibrils. Unlike the results of 2N6S, 2N8S and 2N6,8S, the results of 2N suggested that the binding of the photoacid to insulin fibrils increased the proton transfer rate.

### The spherically-symmetric diffusion model

To extract a quantitative estimation for the kinetic parameters of each photoacid before and after the binding to insulin fibrils, we used the established theoretical paradigm for the excited-state proton transfer of photoacids^11, 16, 29, 30^ where the excited-state radiative decay of the ROH^*^ form is expressed as:

2 $${I}_{f}^{ROH\ast }(t)\cong \frac{\pi {a}^{2}{k}_{a}\exp [-{R}_{D}/a]}{2{k}_{PT}{(\pi Dt)}^{d/2}}$$

where *k*_a_ and *k*_PT_ are the geminate recombination and proton transfer rates, respectively, as described in Eq. (1), occurring on a surface of a reaction sphere with a radius of *a*. *D* is the diffusion coefficient of the dissociated proton, *d* is the dimensionality of the proton diffusion, and *R*_*D*_ is the Debye radius. The Debye radius of photoacids describes the distance where the coulombic attraction between the negative excited-state photoacid after dissociation (*i.e*., the RO^−*^ form), and the positive proton equals the thermal energy (*k*_*B*_ *T*):

3 $${R}_{D}=\frac{|{Z}_{1}{Z}_{2}|{e}^{2}}{4\pi {\varepsilon }_{0}{\varepsilon }_{r}{k}_{B}T}$$

where *Z*_*1*_ and *Z*_*2*_ are the RO^−*^ and proton charges in electron charge units (*i.e*., 1, 2, 2 and 3 electron charge units for 2N, 2N6S, 2N8S and 2N6,8S, respectively, and 1 for the proton), *e* is the elementary electric charge, ε_0_ is the permittivity of free space, ε_r_ is the relative dielectric permittivity of the medium (we used the value of water at 20 °C), *k*_B_ is the Boltzmann constant, and *T* is the temperature. Accordingly, the Debye radii for the used photoacids in the buffered solution are: 7, 14, 14 and 21 Å for 2N, 2N6S, 2N8S and 2N6,8S, respectively.

Krissnel and Agmon^31^ have combined this theoretical kinetic model with the Debye-Smoluchowski diffusion equation for the dissociation probability of an ion-pair (*i.e*., the RO^−*^ … H^+^ pair) at distance *r* and at time *t* to develop a software (SSDP: Spherically-Symmetric Diffusion Problems) for extracting the quantitative values for the described parameters. We used the SSDP (Ver. 2.66) software to examine the change in these parameters for the different photoacids after binding to the insulin fibrillar structure (Table 1 and Figure S2 for the fitted curves). At first, we fitted the curves of the free photoacids in the buffered solution to extract the *a* value, which we estimated to be 4 Å. This value is smaller than the common values for the pyranine photoacid that are in the range of 6–7 Å^16, 23, 32^, which is reasonable due to the smaller molecular size of the 2-naphthol based photoacids. Since the molecules are free in the bulk aqueous solution, we fixed the dimensionality of the proton diffusion to 3, and the diffusion constant (D) to 9 × 10^−5^ cm^2^/s, which is a common value for the diffusion of protons in water. The values that we extracted for *k*_PT_ were: 0.2, 1.0, 1.6 and 1.8 ns^−1^ for 2N, 2N6S, 2N8S and 2N6,8S, respectively. As expected, the values follow the strengths of the different photoacids (see above). The extracted values for 2N and 2N6S are in line with previously measured values^33–37^. The value for 2N8S is 2.5 fold lower than the reported value (~4 ns^−1^)^34, 37^, while the value for 2N6,8S is around an order of magnitude lower than reported values (~17 ns^−1^)^33, 37^. The lower extracted values for 2N8S and 2N6,8S were due to the long pulse width (slow) light source used with a broad instrument response function (IRF), which limited our ability to accurately measure <250 ps lifetimes (see experimental and the IRF curves in Fig. 3). To compensate the effect of the IRF on the fast initial decay of 2N8S and 2N6,8S we re-convoluted the TCSPC decay signal of the photoacids using the IRF function and a 3-exponential decay model (DAS6 software, HORIBA scientific, Figure S3). As stated above, the fast component (initial decay) corresponds to *τ*_PT_. The extracted value (see brackets in Table 1) for 2N8S is in line with the expected one, while the value for 2N6,8S is still lower (less than a factor of 2) than the expected one.

**Table 1:** SSDP fits to the decay of the photoacids before and after the binding to the insulin fibril structure.

		*k*_PT_ [ns^−1^]	*k*_a_ [Å/ns]	*d*	*R*_*D*_ [Å]	D [cm^2^/s]	*a* [Å]
2N	In pH7	0.2	0.2	3	7	9 × 10^−5^	4
	+fibrils	0.4	1.5	1.2	10	5 × 10^−5^	4
2N6S	In pH7	1.0	0.8	3	14	9 × 10^−5^	4
	+fibrils	0.9	0.7	1.9	19	2.5 × 10^−5^	4
2N8S	In pH7	1.6* (3.9)**	1.5	3	14	9 × 10^−5^	4
	+fibrils	0.9	1.4	2.1	16	2.6 × 10^−5^	4
2N6,8S	In pH7	1.8* (10.6)**	1.3	3	21	9 × 10^−5^	4
	+fibrils	0.6	1.2	2.9	22	2 × 10^−5^	4

*These values are underestimated due to the slow light source, see further discussion in the text. **These values are the fast component of a re-convolution of the IRF signal with a 3-exponential decay fit, see further discussion in the text and Figure S3.

Following the binding of the photoacids to insulin fibrils, we observed a number of changes in the extracted parameters:

1. For 2N6S and 2N8S, we found that although the extracted value for *k*_PT_ had decreased following binding to the fibrillar structures, as suggested by the steady-state and time-resolved measurements, this decrease was negligible for 2N6S and by a factor of 2 and 4 for 2N8S (for the extracted values obtained with SSDP and re-convolution, respectively). We also found no significant change in the extracted recombination rates (*k*_*a*_). However, we found that the binding had significantly reduced the diffusion coefficient (D) to ~2.5 × 10^−5^ cm^2^/s, while the dimensionality (*d*) of the diffusion also had a different fractal dimension of ~2. Accordingly, the extracted values suggested that the binding of 2N6S and 2N8S to the insulin fibril structure did not significantly inhibit the proton dissociation; instead, the proton diffused at a much slower rate along the perimeter of the fibril structure (could be visualised as a 2D surface). The slower diffusion was unsurprising due to the partially hydrophobic nature of the structure, which meant the protons could not easily ‘escape’ to the bulk solvent from the surface of the fibril. The partially hydrophobic nature of the structure also induces an increase in the *R*_*D*_ values due to a lower relative dielectric permittivity next to the surface of the insulin structure in comparison to bulk water. The larger increase in the R_D_ values for 2N6S implies that the binding site for it is less water accessible than the one of 2N8S.
2. For 2N6,8S, we found a significant decrease in the *k*_PT_ value by a factor of 3 and 18 (for the extracted values obtained with SSDP and re-convolution, respectively). We found no significant change in both the *k*_*a*_ value and in the dimensionality of the diffusion, but as with findings from previous photoacids, we found a sharp decrease in the diffusion coefficient to a value of 2 × 10^−5^ cm^2^/s. The extracted values suggest that 2N6,8S binds to a relatively hydrophobic pocket on the fibril structure, which dramatically inhibits its ability to release a proton and limits the diffusion of the dissociated protons.
3. For 2N, we found a 2-fold increase in the *k*_PT_ value as qualitatively suggested by the shape of the time-resolved decay. However, unlike the other photoacids, we found a significant increase (9-fold) in the *k*_*a*_ value. We also found that the binding significantly limited the dimensionality of the diffusion to nearly 1D, and although the extracted diffusion constant (5 × 10^−5^ cm^2^/s) was still lower than the one in water, it remained much higher than the values observed for other bound photoacids. This observation suggested that the binding site for 2N on the fibrillar structure enabled an efficient proton transfer in a 1D pathway that subsequently increased the recombination rate of the proton.

### Molecular Dynamics simulations

**Figure 4:**
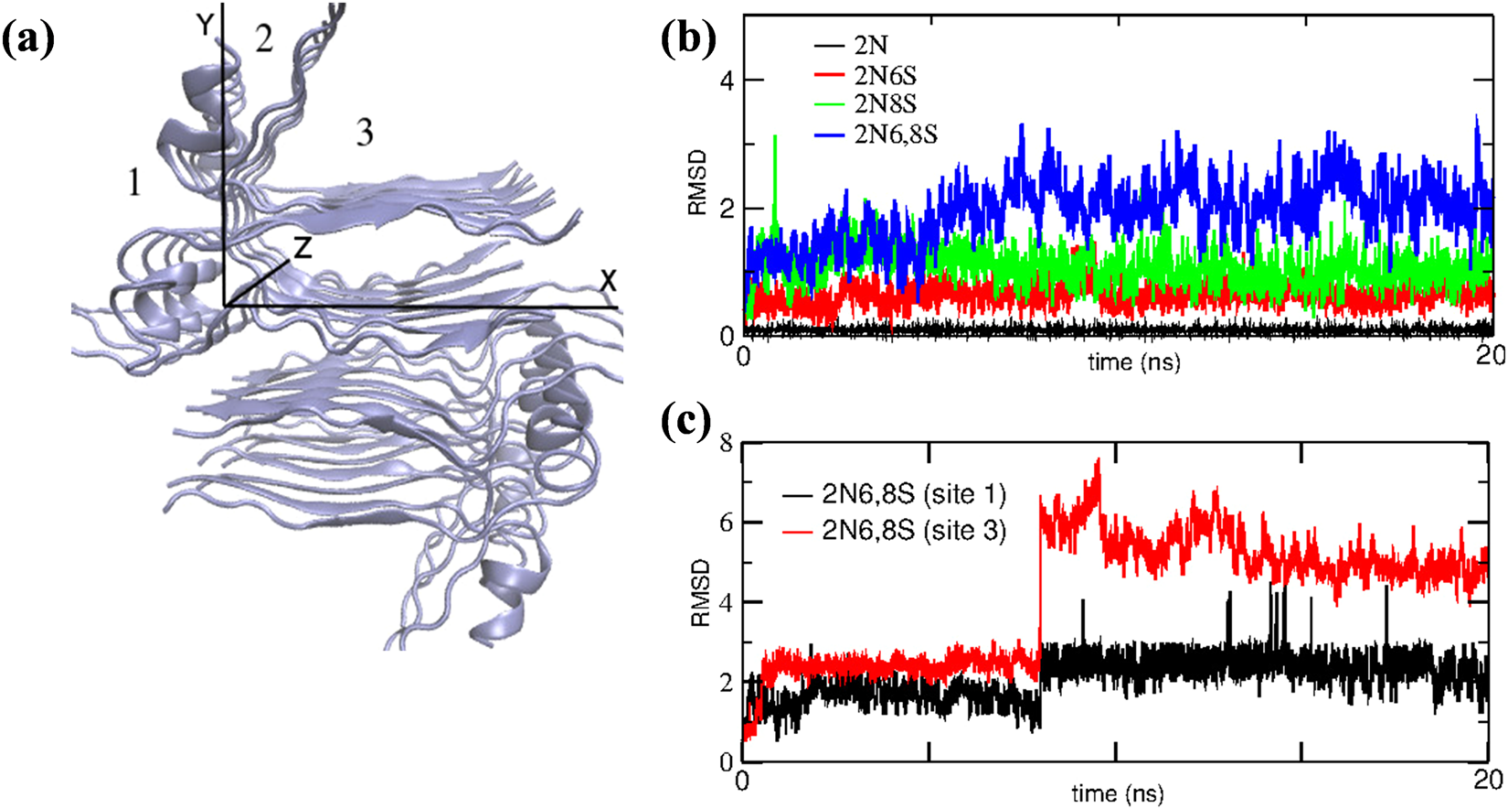
(**a**) The modelled structure of the insulin fibril. Essential axes shown include the 3D coordinate axes (X, Y, Z); the fibril axis is along the Z axis. (**b**) Positional fluctuations of each photoacid as a function of time in binding site 2 and (**c**) of 2N6,8S in binding sites 1 and 3. The large ‘jump’ in the trace for 2N6,8S after ~7.5 ns in binding site 3 is due to changes in the binding mode of 2N6,8S to the protein surface in this binding site (see structures and discussion in Figures S5–S8).

In order to test our hypothesis for the different binding modes of the various photoacids, we employed MD to model the surface of the insulin fibril and examine the possible binding sites for the photoacids. The results of Ivanova *et al*.^38^ informed our surface model of the insulin fibril (Fig. 4a). We further confirmed that the structures obtained in acidic pH (as the one of Ivanova *et al*.) are comparable to the structures at neutral pH7 (Figure S4 and text within). We used eight asymmetric units of the fibril model to achieve a 35 Å pitch, which was large enough to study the binding of the photoacids to the fibril surfaces, where the photoacids could bind to all the accessible solvent surface area. Using a molecular docking algorithm followed by all-atom MD simulations (see experimental), we identified and characterised three binding sites for the photoacids, which are marked as 1, 2 and 3 in Fig. 4a. By comparing these three binding sites, we found that all of the studied photoacids could bind stably to binding site number 2 (Fig. 4b). The 2N6,8S photoacid also bound stably to binding sites 1 and 3 (Fig. 4c, see also further discussion and Figures S5–S7 in the supporting information), while other photoacids washed out during a 20 ns simulation (Figure S8). We extended the simulations in binding sites 1 and 3 to 40 ns which confirmed the stable binding of the 2N6,8S (Figure S9). We further found that the binding modes of different photoacids to binding site 2 could vary. Specifically, as shown in Fig. 4b, 2N binds very strongly and was buried in the structure of the binding site with minimal fluctuations as a function of time, while the other photoacids fluctuated much more when bound to this site. An additional indication for the location of the photoacid in binding site 2 can be visualized by the distribution of angles we used to characterise the mutual orientation between the photoacid and the fibril (Figure S10 and text within).

**Figure 5:**
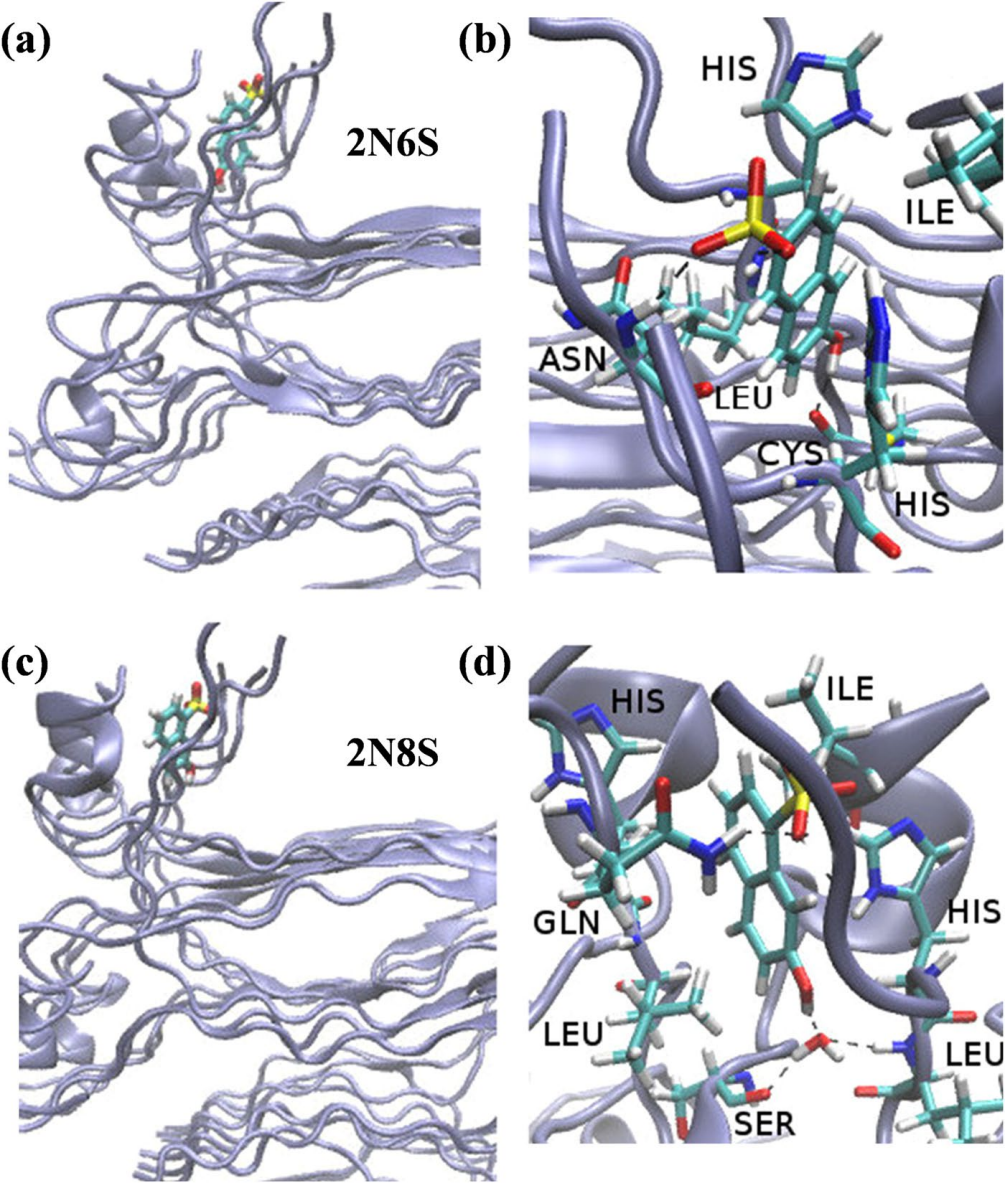
A bird’s-eye view of (**a**) 2N6S and (**c**) 2N8S bound to binding site 2 on the insulin fibril surface. (**b** and **d**) Close-up view of (**a**) and (**c**), respectively.

The magnitude of the fluctuations was also an indicator for each molecule’s reorientation ability. This magnitude was comparable for 2N6S and 2N8S, although 2N8S appeared to fluctuate slightly more than 2N6S. This was likely due to the more accessible sulfonic group positions of 2N6S that could form a H-bond with the amide backbone of the protein, which made it slightly less mobile (Fig. 5a and b). We also observed that 2N8S can form a relatively stable H-bond with water molecule (Fig. 5c and d). Such a water bridge provides the photoacid with some additional degrees of orientation freedom, which might explain its higher level of fluctuation and the larger orientational angle distribution in the bound state (Figure S10). In addition to the relatively stable water molecule binding to 2N8S (for more than 10 ns of the simulation) in binding site 2, we observed water molecules entering the binding site to transiently interact with 2N6S. The stable and transient interactions between the hydroxyl of 2N6S and 2N8S and water molecules support our theory that the diffusion of protons from 2N6S and 2N8S is mediated by water. The naphthol group of 2N6S and 2N8S (Fig. 5a and c) was also positioned parallel to the fibril surface, *i.e*., could be visualised as lying down on the surface.

**Figure 6:**
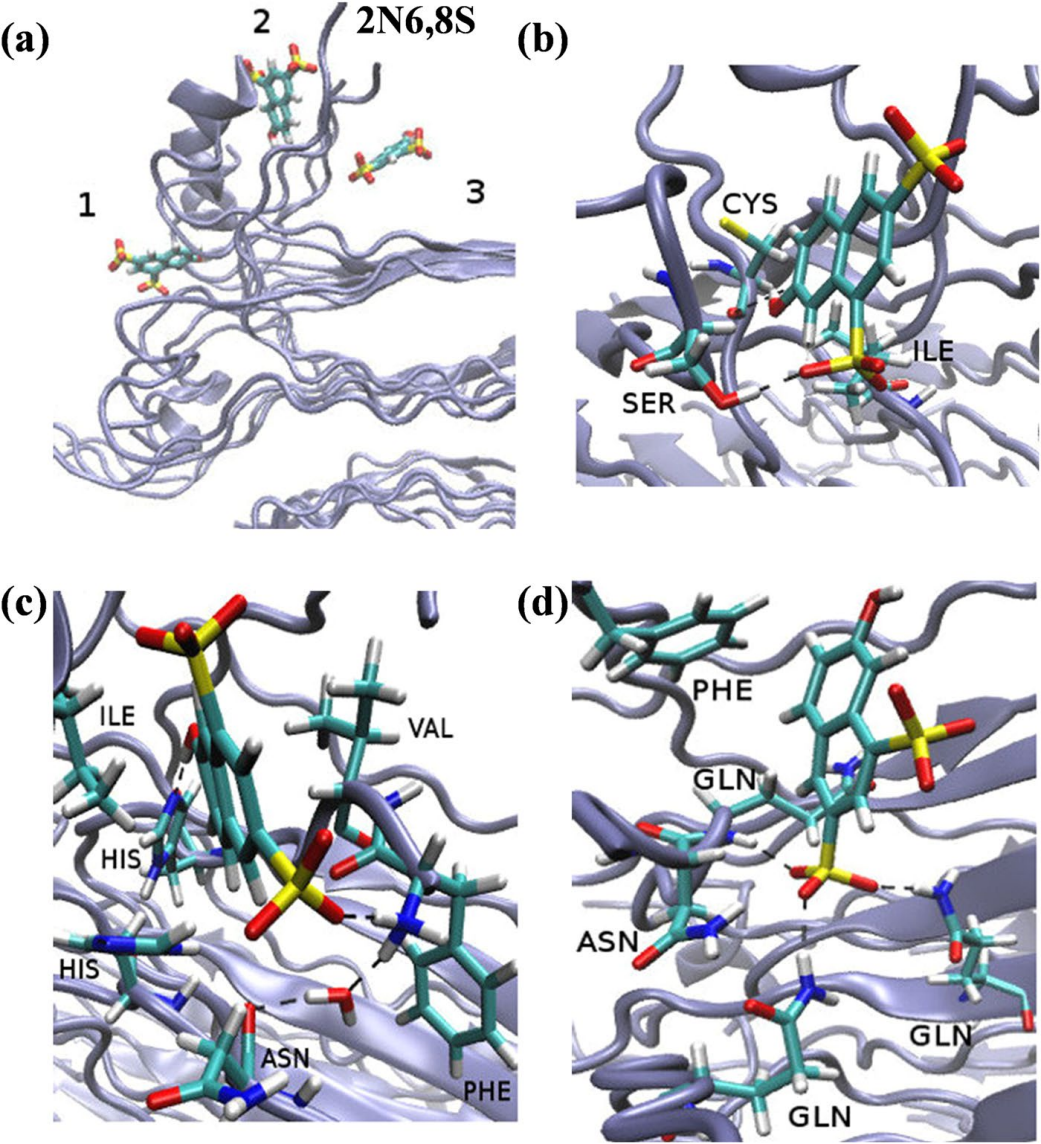
(**a**) A bird’s-eye view of 2N6,8S at the different binding sites on the insulin fibril surface. The close-up view of 2N6,8S in binding sites (**b**) 1, (**c**) 2, and (**d**) 3.

The fluctuation of 2N6,8S in binding site 2 (Fig. 6b) had the largest magnitude, which was mainly due to its bulkier molecular size, but also because it formed a water mediated (rather than direct) H-bond inside the hydrophobic core (Fig. 6c). It should be noted that in the other binding sites (Figs 6b and 6d), 2N6,8S fluctuated within the same order of magnitude (Fig. 4c), albeit with a re-orientation in its binding geometry because of ion interruptions, whereas ions were less accessible in binding site 2. The position of 2N6,8S (Fig. 6a) in all of the binding sites was roughly in the middle of the site due to steric interruption of the two sulfonic groups. However, due to the more hydrophobic nature of all the binding sites compared to bulk solution, the proton transfer rate of 2N6,8S was significantly reduced following binding to the insulin fibril structure.

**Figure 7:**
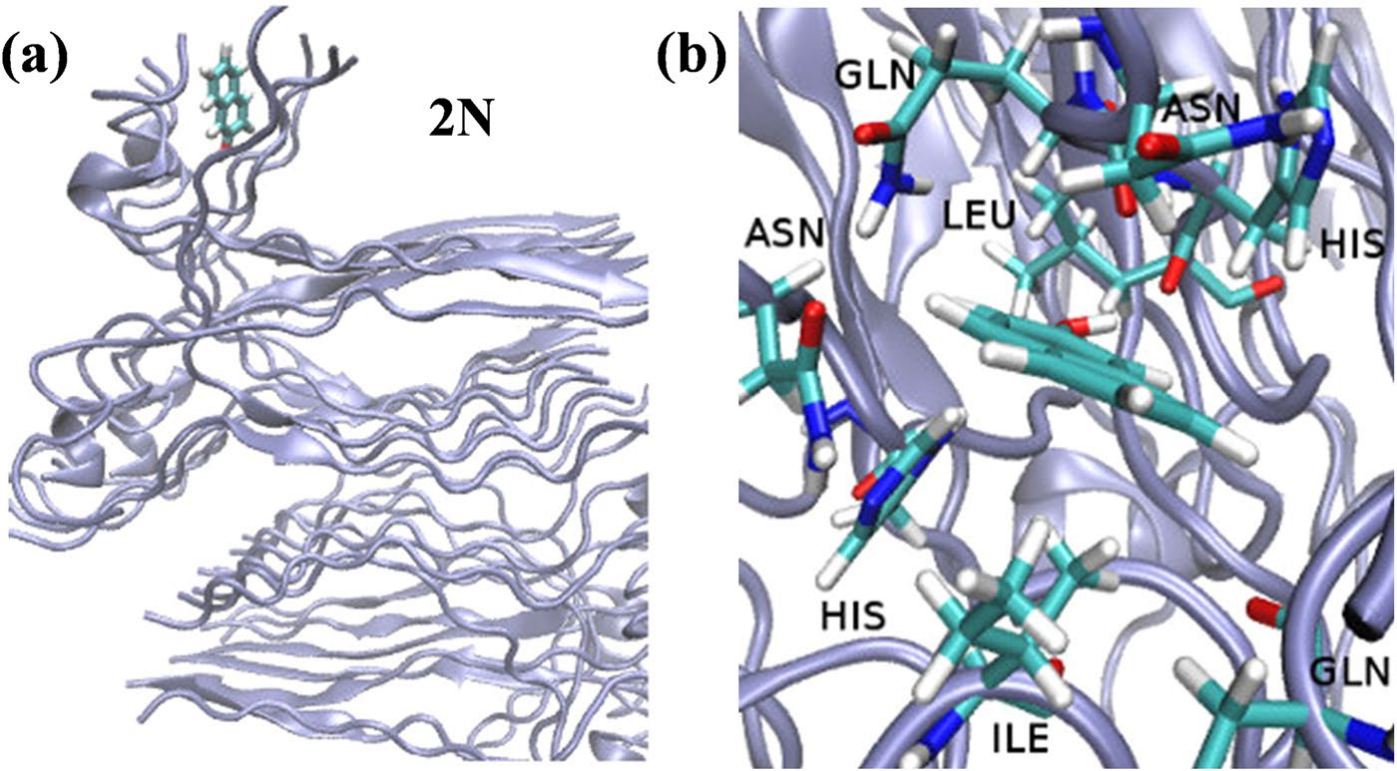
(**a**) A bird’s-eye view and (**b**) close-up view of the binding mode of 2N to binding site 2 on the insulin fibril surface.

We found that the strong binding of 2N to the fibril structure, which resulted in minimum fluctuations of this photoacid in compared to others (Fig. 4b), was assisted by both hydrophobic and hydrophilic interactions. Together with the absence of steric interruption of sulfonic groups as seen in other photoacids, 2N was able to permeate deep into the pocket created by binding site 2. The fixed position of 2N in this binding site (Fig. 7) was between two His residues, with a very close (<3 Å) distance between the hydroxyl of 2N to one of the His residues. This intimate interaction between 2N and His might explain our observation whereby the His residue serves as the proton acceptor for the photoacid while the fixed position of this acid-base dictates a 1D directionality, and can also substantially increase the recombination process. Support for our explanation can be found in the natural role of His to serve as a proton shuttle in enzymatic catalysis^39^. Since the hydroxyl of 2N was buried in the insulin fibril structure, we did not find transient H-bonds between it and water molecules, which supported our theory that the diffusion from 2N was not water-mediated.

### Following the amyloidogenesis process using photoacids

The ability to follow the amyloidogenesis process is one of the most important aspects in the study of amyloid fibrils. The current common methodology is the use of fluorescent probes, where the most common one is thioflavin T (ThT)^40–42^, and various other fluorescent molecular probes have been suggested as well^43–46^. Though the differences in the molecular structure of the different fluorescent probes for detecting amyloid fibrils are large, they all share the same photophysical origin for their increase in fluorescence upon binding to amyloid fibrils due to their molecular rotor behaviour^47^. In this mechanism, a nonradiative charge transfer state between the locally excited state of the molecule and the ground state takes place at a defined structural configuration of the molecule, usually described by a certain bond angle and referred to as a twisted intramolecular charge transfer (TICT) state. The fast rotation of the molecule when it is free in solution results in frequent TICT events, hence the fluorescence quantum efficiency of the probe is very low. However, upon binding to amyloid fibrils, the free rotation of the molecule is inhibited, which induces an increase in the fluorescence efficiency.

**Figure 8:**
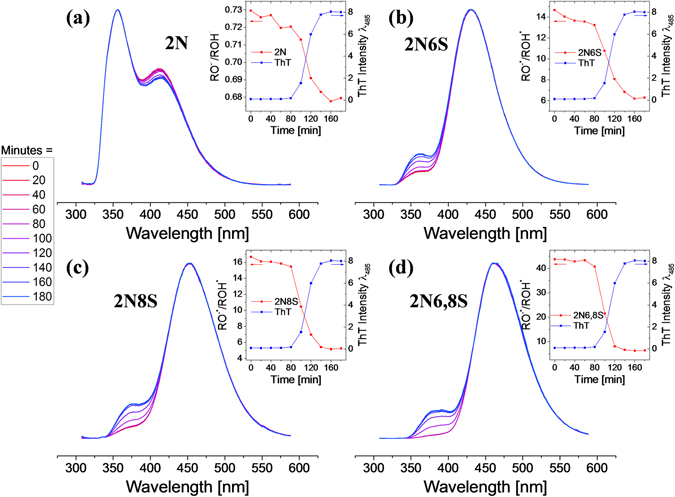
Normalized steady-state fluorescence of the different photoacids: (**a**) 2N, (**b**) 2N6S, (**c**) 2N8S and (**d**) 2N6,8S during the amyloidogenesis process at 20 min intervals from 0 min (red) to 180 min (blue), together with kinetics traces (inset) comparing the RO^−*^/ROH^*^ ratio of the photoacid with ThT emission as a function of time.

As described above, the photophysical origin for the change of fluorescence in photoacids is completely different. Rather than relying on the fluorescence efficiency of the probe, the RO^−*^/ROH^*^ ratio is being probed in the steady-state measurements. We compared the change in the steady-state emission of the photoacids during the amyloidogenesis process (Fig. 8) to ThT emission (Figure S11). Steady-state measurements are the common practice to follow the amyloidogenesis process with ThT either by using a fluorescent plate reader or a fluorometer, as was done in this study. The kinetic profiles of the amyloidogenesis process obtained by the use of photoacids correlate well with the one obtained by the use of ThT (inset of Fig. 8), comprising a lag phase followed by a rapid exponential growth until the stationary stage. This finding not only validates our above results but also suggests that photoacids can be used as fluorescent markers for following the amyloidogenesis process. Moreover, while ThT acts as an on/off switch and its radiative emission “lights up” only upon binding to the amyloid structure, the RO^−*^/ROH^*^ ratio of photoacids is more sensitive to the hydrated environment of the probe, hence they can be used as markers for following even small structural transitions of proteins^25^. It has been well established that insulin (as well as other amyloidogenic proteins) goes through several structural intermediates before the formation of the mature fibril^48–50^. Due to the on/off fluorescent activity, ThT is not efficient in observing the intermediates in the lag phase (Figure S14), though it is important to stress that other molecular rotors have been argued to have better sensitivity than ThT in observing amyloid intermediates^46^. On the other side, a noticeable decrease in the RO^−*^/ROH^*^ ratio of the photoacids can be observed during the lag phase (Figs 8 and S12). This decrease suggests the formation of a binding site on the surface of insulin that becomes less water accessible as a function of time, most probably due to the formation of higher-hierarchy structures (oligomers). Importantly, we did not observe significant changes in the steady-state spectrum of the photoacid with or without insulin monomers (at t = 0 minutes), and we can conclude that the monomer itself does not have a binding site for the photoacid. The relatively weak binding of 2N6,8S, together with the water accessibility to the binding site occupied by 2N6,8S, makes this photoacid less efficient in comparison to the other photoacids in observing minor structural changes of insulin during the lag phase. However, due to its highest difference in the RO^−*^/ROH^*^ fluorescence ratio between the unbound and fibril-bound states, 2N6,8S will be a better probe for fibrils in comparison to the rest of the photoacids studied in this work. To conclude, while the ThT assay is useful for following the aggregation and fibrillization of amyloid proteins, we suggest that photoacids can be used as well to gain better understanding on the environment of the binding site.

## Experimental Section

### Insulin fibrils formation

Gibco^®^ recombinant (animal-origin-free) human insulin (Life Technologies) was dissolved in 150 mM NaCl pH 1.6 at a final concentration of 2.5 mg/ml. The solution was placed in 65 °C for 5 h for the formation of fibrils, where the solution has transformed from a clear solution to an opaque one, which is a clear indication for the formation of aggregated fibrillary structures. In order to change the buffer to pH7, the insulin fibril solution was spun down at ~10,000 rpm for 2 min, and the supernatant was replaced by 4 times diluted PBS buffer (final ion concentration ~35 mM NaCl, 0.7 mM KCl, 2.5 mM Na_2_HPO_4_ and 0.5 mM KH_2_PO_4_), adjusted to pH7.

### Steady-state and time-resolved fluorescence measurements

A stock solution of 10 mM of 2-naphthol-6-sulfonic acid potassium salt (2N6S, Sigma-Aldrich), 2-naphthol-8-sulfonic acid potassium salt (2N8S, TCI chemicals), and 2-naphthol-6,8-disulfonic acid disodium salt (2N6,8S, Sigma-Aldrich) was prepared in deionised water, while a stock solution of 2-naphthol (2N, Sigma-Aldrich) was prepared in 25% ethanol in deionised water (due to the lower solubility of 2N). A 4.5 μl aliquot from the stock solution was added to a 1 ml buffer solution, with or without the insulin fibrils, for a final photoacid concentration of 45 μM. The photoacid-insulin fibril solution was placed in room temperature with mild shaking for at least 1 h to allow the binding of the photoacid to the fibrillar structure. Following incubation, the latter solution was spun down at ~10,000 rpm for 2 min, and the supernatant was replaced by the above PBS buffer. A Fluorolog system (Horiba) with 1 nm bandpass slits in both the entrance and exit arms was used for the steady-state measurements and the sample was excited at 300 nm. A Deltaflex system (Horiba) was used for time-resolved emission spectroscopy with a 282 nm light emitting diode (1 ns nominal pulse duration with 1 MHz repetition rate) as the excitation source. The emission (>10,000 counts at peak) was collected at 350 nm for 2N, 355 nm for 2N6S and 2N8S, and 370 nm for 2N6,8S. The instrument response function was collected by a diluted Ludox© solution (Sigma-Aldrich) in water at the excitation wavelength.

### Steady-state fluorescence measurements of the amyloidogenesis process

A stock solution of 10 mM of thioflavin T (ThT, Sigma-Aldrich) was prepared in deionised water. A 30 μl aliquot from the 10 mM stock solutions of 2 N, 2N6S, 2N8S, 2 N6,8S or ThT was added to 15 ml of the above pH7 PBS solution to receive a 20 μM solution of the fluorescent molecule. Human insulin was dissolved in 7 ml of 150 mM NaCl pH 1.6 at a final concentration of 2.5 mg/ml and was placed in 65 °C. At 20 minutes intervals, 60 μl from the insulin solution was placed in 540 μl of the fluorescent molecule solution. The fluorescence steady-state was monitored with the above Fluorolog system with excitation wavelengths of 300 nm for the photoacids and 440 nm for ThT.

### Molecular dynamics (MD) simulation

The insulin fibril model was based on experimental data from Ivanova *et al*.^38^ and built with eight asymmetric units in which each unit was composed of two insulin molecules and translated by 4.74 Å with a twist of 0.71° around the fibril axis. The structure was solvated in a periodic box with dimensions [10.4 × 3.5 × 9.0] nm^3^ with TIP3P^51^ water and 40 mM NaCl (experimental value). The system was neutralised with counter ions, reaching the total size of ~29,000 atoms. The protein fibril geometry was then relaxed in two steps: (i) side chains released while keeping the backbone restrain of 10 kcal/mol/Å^2^, and (ii) backbone (the whole protein fibril) structure released. The harmonic restrain of 5, 3, 1, 0.5 and 0.1 kcal/mol/Å^2^ descending steps were used during the side chain and backbone (whole protein) release, with a total of 20 ns MD carried out to relax the fibril structure in solvent. The whole protein was further relaxed for another 80 ns MD simulation to fully equilibrate the free fibril structure in solution. The fibril geometry at every 10 ns from 80 ns MD simulation was saved to use for ligand (photoacids) docking.

We employed the program HADDOCK (version 2.2)^52^ for protein-ligand complex structure prediction. The docking program was set to enable the photoacids to approach the surface ~2 Å from any solvent accessible fibril residue. During the docking refinement stage, protein side chains were kept flexible and the photoacid was fully flexible.

The most stable docked structure of the photoacids on three surfaces was used to create a single photoacid-fibril complex by simultaneously placing the photoacid (of one type) in each of the three binding sites using the molecular building and visualisation software VMD^53^. The complex with all 3 binding sites occupied by a certain photoacid was then used for 20 ns MD refinement simulations for each type of photoacid without any constraints in aqueous solvent.

All complex simulations were carried out with NAMD software (version 2.9)^54^ using CHARMM 36 parameters^55, 56^. Photoacid parameters were obtained using the CHARMM general force field algorithm implemented in the CGenFF program^57, 58^. Because PA dyes are quite rigid, we only optimised the partial charges at MO62X/6-31G(d) level with PCM in SCRF continuum models^59, 60^. MD simulations were performed in NPT ensemble with the temperature and pressure maintained at 300 K and 1 atm, respectively, via Langevin coupling with damping coefficient of 5 ps^−1^. All bonds to hydrogen atoms were maintained using the SHAKE algorithm^61^. Periodic boundary conditions used the particle-mesh Ewald^62^ algorithm to compute the long-range electrostatic interactions. Lennard-Jones (LJ) potential was switched off in between the switching distance and the cutoff (10–12 Å) using a force-switching function. Non-bonded pair list cutoff of 14 Å was used and a 2 fs time step was maintained throughout the simulations.

## Conclusions

In summary, we have shown that photoacids bind differently to the insulin fibril surface by steady-state and time-resolved emission measurements. Our experiments are supported by theoretical analyses of the results and all-atom MD simulations. The photoacids we examined differ by the number and location of their sulfonic groups, which control the proton transfer efficiency of each photoacid. We found that following binding to the insulin fibril, the excited-state proton transfer properties of each photoacid changed in a different manner. For the strongest photoacid of 2N6,8S, we found that the binding decreased the proton-transfer rate by at least 3-fold, and although the proton diffusion was still in 3D, its diffusion coefficient was much slower than in bulk solution. For the photoacids of 2N6S and 2N8S, we found that the binding did not significantly change their kinetic rates, but the dimensionality of the diffusion had been reduced to 2D and was much slower than in water. For the weakest photoacid of 2N, we found that binding to the fibrillar structure increased both the proton transfer rate and the geminate recombination rate by 2- and 9-fold, respectively, and that the proton diffusion had a fractal dimensionality of nearly 1D. Using MD, we were able to gain insight into the molecular details of specific location and binding mode of each photoacid along the fibrillar structure. We found that 2N6,8S was not positionally restrained in its binding sites and fluctuated quite intensely, which did not affect the dimensionality of the proton diffusion (remained 3D). However, since it is a strong photoacid, the relative hydrophobicity of the insulin fibril surface compared to water significantly reduced its proton-transfer rate. The MD study also suggested that 2N6S and 2N8S were weakly bound closer to the entry to the binding pocket. The loose binding of 2N6S and 2N8S to the fibril structure permitted the entrance of water molecules into the binding site, which could transiently interact with the hydroxyl of the photoacid. This transient interaction between water molecules and the photoacid suggested that the proton diffusion from 2N6S and 2N8S was mediated by water. Perhaps the most interesting finding is that 2N bound very strongly and it was buried within the structure between two His residues. We hypothesised that the close proximity between 2N and the His residue observed in the MD simulations results in a proton transfer between 2N and the His. This explains the efficient proton-transfer rate, the high geminate-recombination rate, and the 1-dimensionality of the diffusion. We further used all of the photoacids for following the amyloidogenesis by comparing them to the most used molecular probe of ThT, where we received similar kinetic curves for the process, hence implying that (i) they can be used as markers, and (ii) that the observed changes in the fluorescence properties are indeed due to the binding to the fibrils.

## Electronic supplementary material

Supplemental Figures
