# Supplementary material for: Exploring the binding sites and proton diffusion on insulin amyloid fibril surfaces by naphthol-based photoacid fluorescence and molecular simulations
Source: Sci Rep. 2017 Jul 24;7:6245. doi: 10.1038/s41598-017-06030-4 (PMC5524688; doi:10.1038/s41598-017-06030-4)
Supplement: Supplementary file 1 — Supplemental Figures [file 41598_2017_6030_MOESM1_ESM.pdf]

Supplementary Information:

Exploring the binding sites and proton diffusion on insulin amyloid fibril surfaces by naphthol-based photoacid fluorescence and molecular simulations

Nadav Amdursky,[1,3,*] M. Harunur Rashid,[2] Molly M. Stevens,[1] Irene Yarovsky[2,*]

[1]*Department of Materials, Department of Bioengineering and Institute of Biomedical Engineering, Imperial College London, London, SW7 2AZ, United Kingdom*

[2]*School of Engineering, RMIT University, Melbourne, Victoria, 3001, Australia*

[3]*Schulich Faculty of Chemistry, Technion - Israel Institute of Technology, Haifa, 3200003, Israel.*

e-mails: amdursky@technion.ac.il; irene.yarovsky@rmit.edu.au

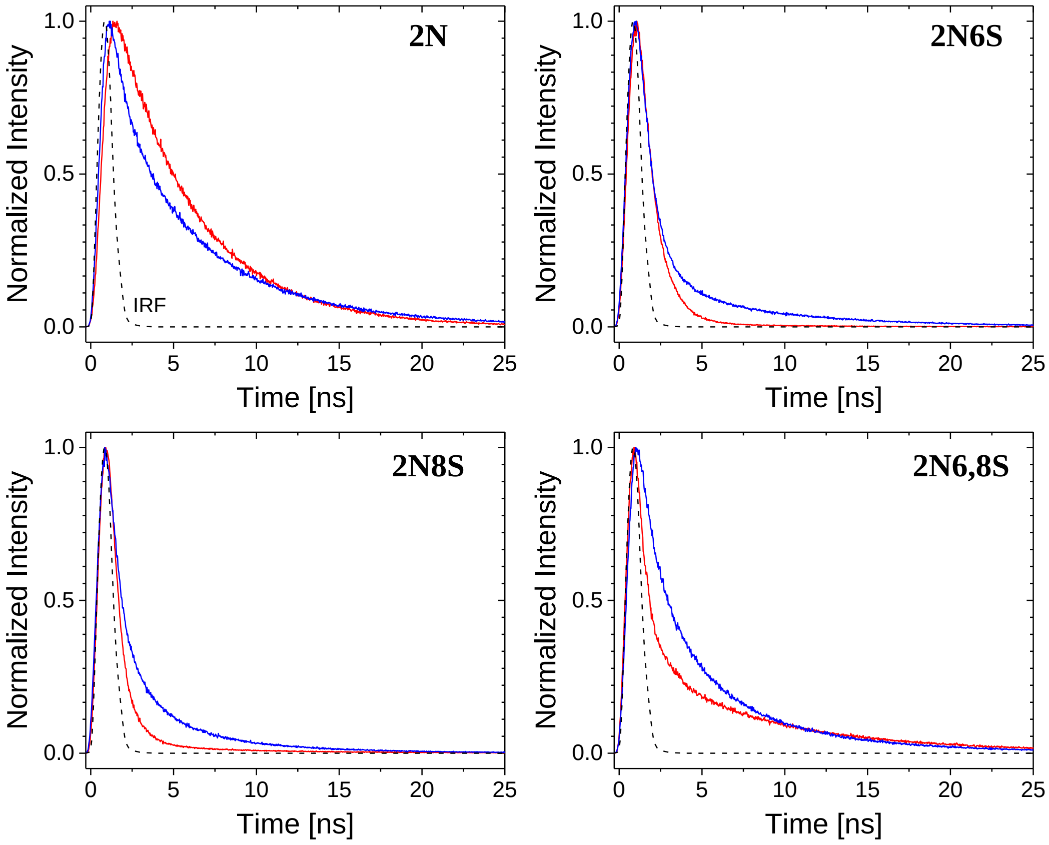

Figure S1. Time-resolved fluorescence on a linear scale of the different photoacids in pH7 buffer (red curves), and in the same buffer with insulin fibrils (blue curves), together with the instrument response function (IRF, dashed black curves).

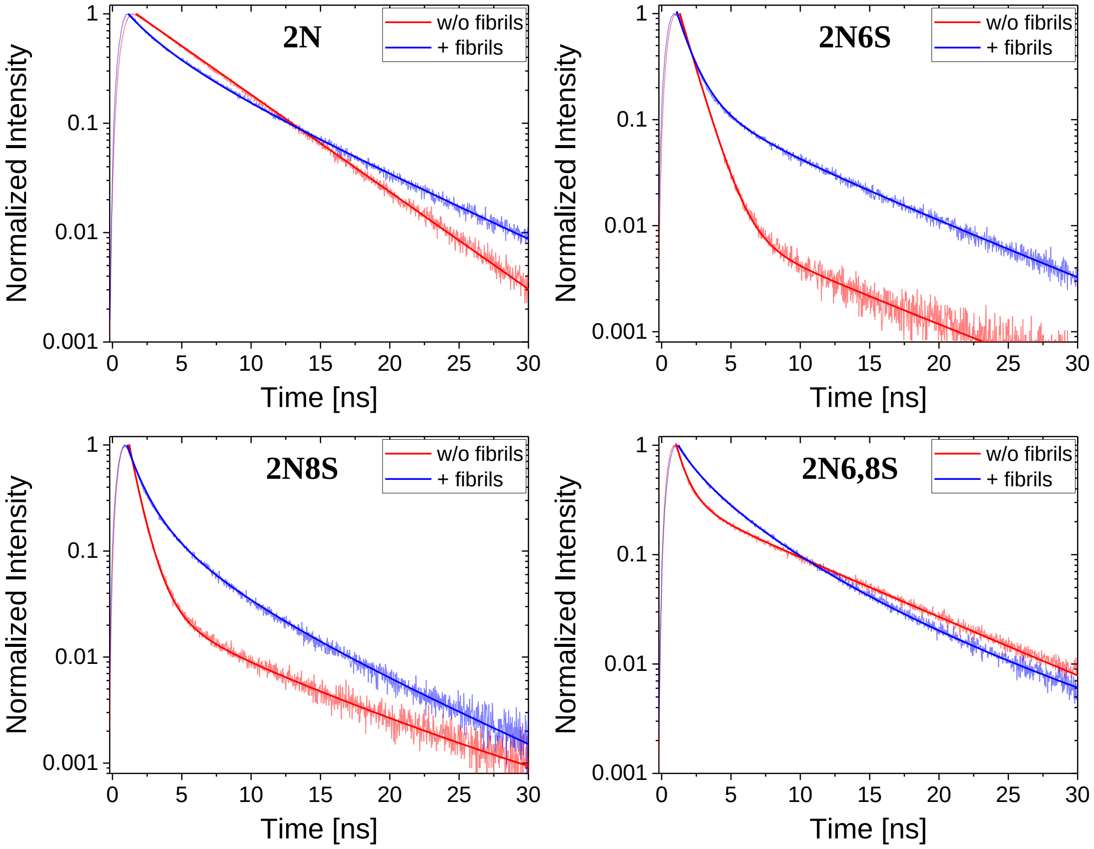

Figure S2. The SSDP fits for the decays presented in Figure 2.

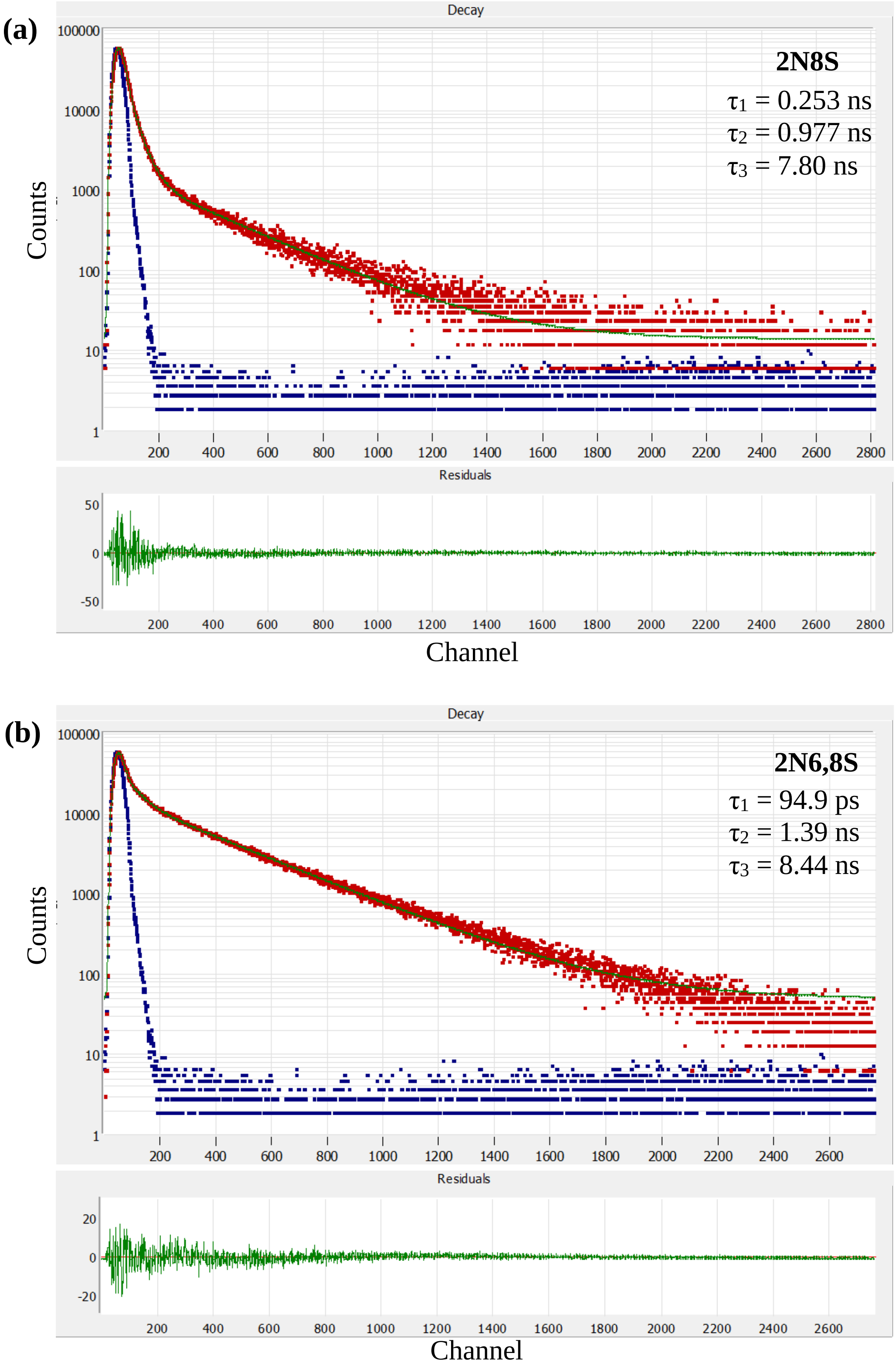

Figure S3. A re-convoluted fit of the TCSPC signals for (a) 2N8S and (b) 2N6,8S in pH7 buffer using a 3-exponential decay fit. The red and blue signals are for the photoacid and the IRF, respectively.

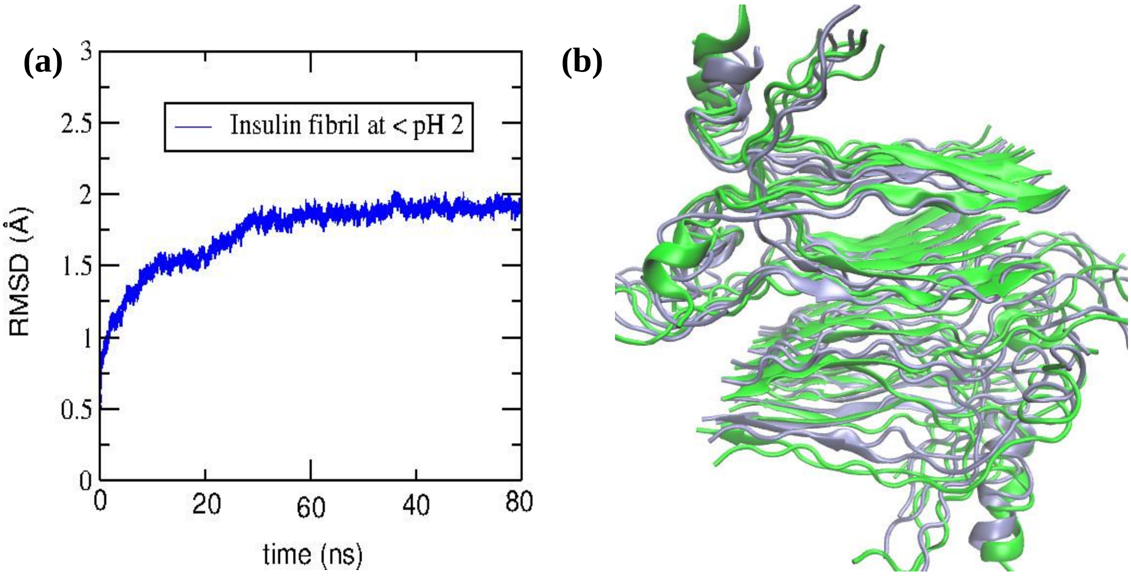

Figure S4. (a) RMSD of backbone heavy atoms of insulin at pH2 relative to the last frame of 80 ns insulin fibril at pH 7. (b) Superimposed insulin fibrils at pH2 (grey) and pH7 (green).

To explore any possible structural changes of the model as the fibril environment changed from the acidic to neutral pH we performed the 80 ns MD simulation of the fibril structure at pH7 and at pH2. We found that the RMSD of the insulin fibril structure at pH2 changed only by ~2 Å relative to the fibril at pH7 (Figure S4a). We also aligned the final frames of the simulated fibril structures at pH2 and pH7 and found no significant differences in the backbone orientation (Figure S4b).

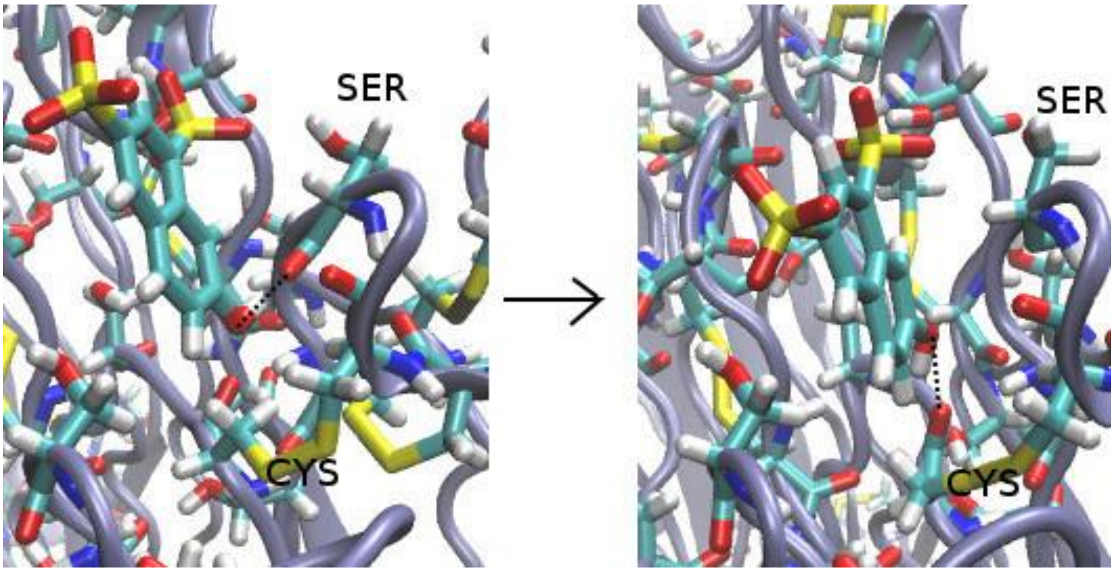

Figure S5. **Binding of 2N6,8S to binding site 1**. Initially, 2N6,8S formed a hydrogen bond with the Ser carbonyl oxygen (dotted line in left panel) in binding site 1. After ~7.5 ns of MD equilibration the molecule enters deeper into the fibril surface to form a new hydrogen bond with the Cys carbonyl oxygen (dotted line in right panel) accompanied by multiple VdW interactions that enabled stronger binding. The hydrogen bond distances are plotted in Figure S7.

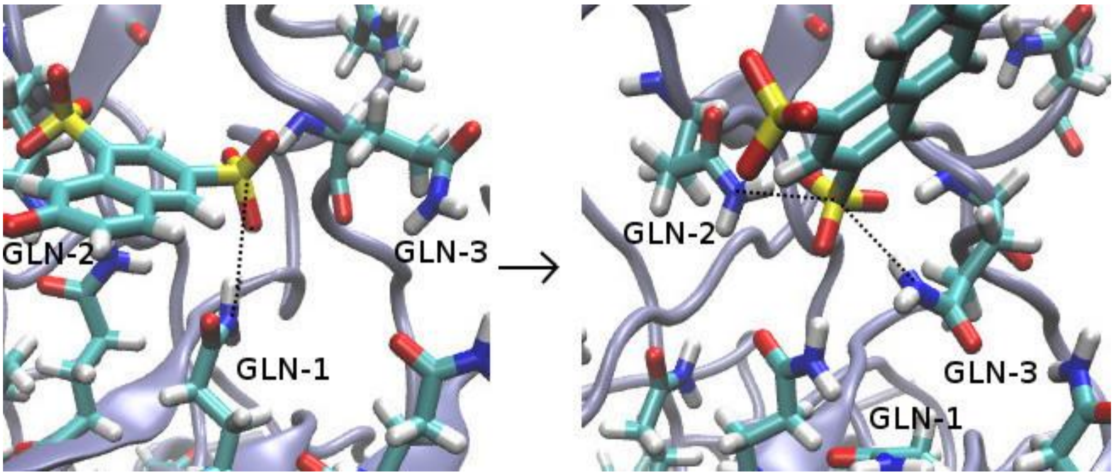

Figure S6. **Binding of 2N6,8S to binding site 3**. Initially, 2N6,8S binds within a pocket lined up by three Gln residues in binding site 3. After ~7.5 ns of MD simulation the H-bond between the molecule and Gln-1 (dotted line, left panel) was replaced by a stronger binding mode with two H-bonds formed with Gln-2 and Gln-3 residues. All of the distances are plotted in Figure S7.

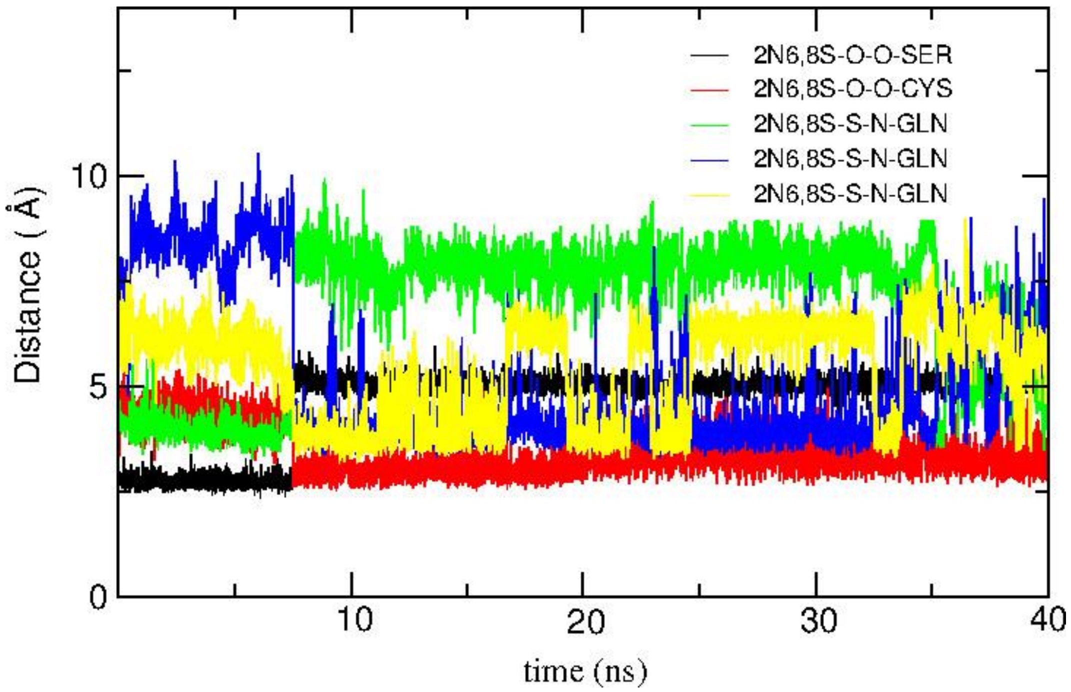

Figure S7. 2N6,8S interaction distances with Ser (black), Cys (red), Gln-1 (green), Gln-2 (blue) and Gln-3 (yellow) residues identified in Figures S5-S6.

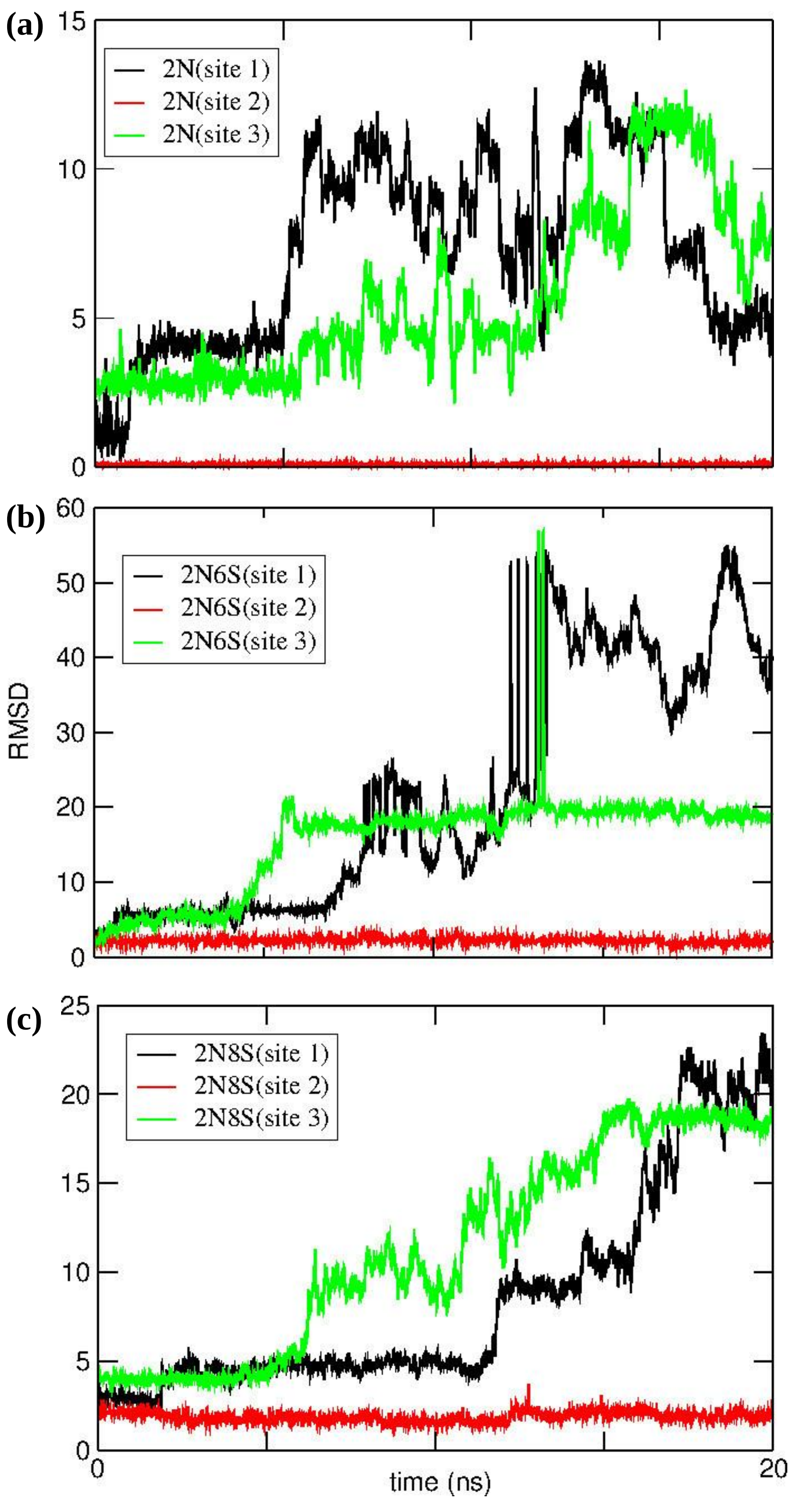

Figure S8. Positional fluctuations of (a) 2N, (b) 2N6S and (c) 2N8S in binding sites 1, 2 and 3 as a function of time, indicating the loss of interaction between the photoacid and the fibril surface in binding sites 1 and 3 within < 5 ns.

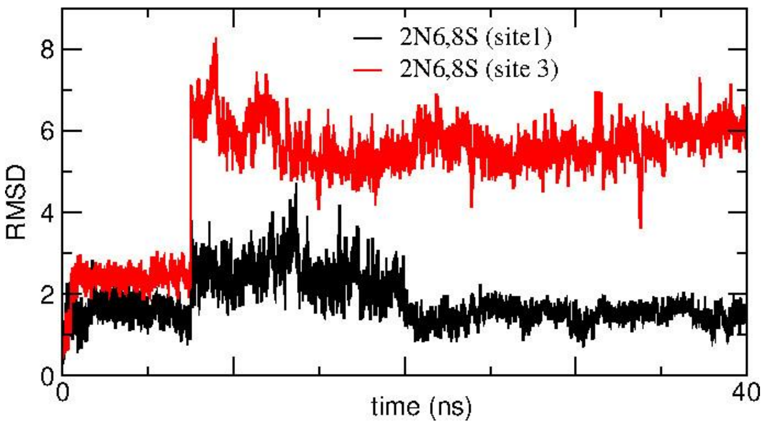

Figure S9. Positional fluctuations of 2N6,8S in binding sites 1 and 3 as a function of time, extended to a 40 ns time frame, indicating the stable binding of 2N6,8S in these binding sites.

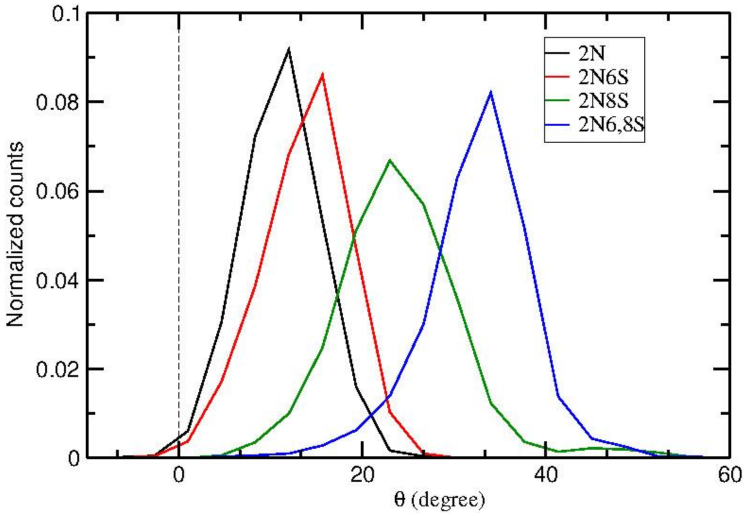

Figure S10. The distribution of angles (θ) between the photoacid plane and the Y axis plotted for all photoacids in binding site 2 (see Figure 4a for axes definition).

Figure S10 shows the distribution of angles (θ) between the photoacid plane (defined as the plane of the conjugated rings) and the Y axis in and around the narrow hydrophobic core of binding site 2. 2N went deeply inside the hydrophobic core as reflected by the narrow distribution around the small angle values (black line), which could also be used as a reference for identifying the location of the other photoacids with respect to the narrow hydrophobic core of this binding site. The larger angle values indicated that the photoacid was not located deep inside the pocket (due to the volume restrictions) and was instead located closer to the pocket entry as seen progressively for the larger 2N6S (red), 2N8S (green), and the biggest 2N6,8S molecule (blue). The angle distribution was wider for more significantly fluctuating molecules located closer to the fibril surface (entrance to the pocket).

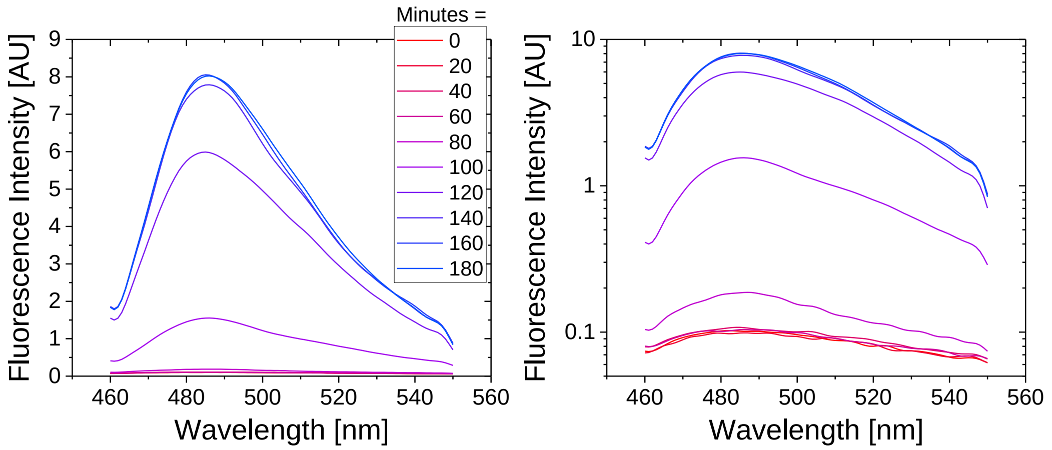

Figure S11. ThT steady-state emission during the amyloidogenesis process on a linear and semi-logarithmic scales (left and right panels, respectively)

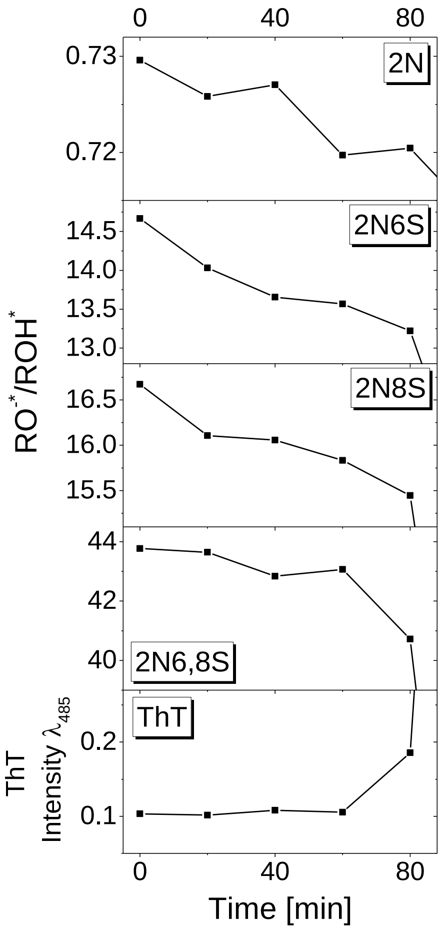

Figure S12. Kinetics traces of the lag phase of the amyloidogenesis process comparing the RO^{-*}/ ROH^{*} ratio of the photoacids with ThT emission as a function of time.
